# Prefecture-level factors associated with home-visit dental care utilization in Japan: focus on dentist workforce characteristics

**DOI:** 10.1186/s12903-026-08914-2

**Published:** 2026-06-20

**Authors:** Emina Masuda, Kazuo Fujita, Akiyoshi Sekoguchi, Keisuke Nomura, Hideki Yamamoto, Hideto Takahashi

**Affiliations:** 1 Japan Dental Association Research Institute, 4-1-20 Kudankita, Chiyoda-ku, Tokyo, 102-0073 Japan; 2 Japan Dental Association, 4-1-20 Kudankita, Chiyoda-ku, Tokyo, 102-0073 Japan

**Keywords:** Aging, Dental care, Dentists, Healthcare disparities, Home care services

## Abstract

**Background:**

Population aging has increased demand for home-visit dental care in Japan, yet regional disparities persist, as in other aging societies. This study aimed to identify factors associated with regional variation in the utilization of home-visit dental care. As part of an exploratory approach, we also examined home-visit medical care data to consider possible contrasts with dental service provision.

**Methods:**

This ecological cross-sectional study followed the STROBE guidelines and used prefecture-level aggregated data. We calculated the standardized claim ratio (SCR) for home-visit dental services using the 9th edition of the National Database of Health Insurance Claims and Specific Health Checkups of Japan (NDB) Open Data (April 2022 to March 2023). Spearman’s correlation coefficient was computed to examine associations between SCR and relevant variables, including average age of dentists, number of dental clinics, and number of dentists in medical facilities. Then, multiple regression analyses were conducted using the SCR of the home-visit dental services, adjusting for the average age of dentists, the number of dental clinics, and the regional aging rate.

**Results:**

Prefectural SCRs for home-visit dental services varied widely (range 24.68–168.79, median 70.52). SCR was negatively correlated with the average age of dentists (R = − 0.65, *p* < 0.001). In addition, it was positively correlated with the number of dentists working in medical facilities per 100,000 population (R = 0.60, *p* < 0.001) and number of dental clinics per 100,000 population (R = 0.71, *p* < 0.001). Multiple regression analysis showed that the average age of dentists had a negative relationship with SCR; the standardized regression coefficient (β) was − 0.30 (*p* = 0.017), whereas the number of dental clinics had a positive relationship (β = 0.47, *p* < 0.001).

**Conclusion:**

Regional variation in home-visit dental care utilization was associated with structural characteristics of the dental workforce. However, these findings should be interpreted as ecological associations influenced by both supply- and demand-side factors, rather than as indicators of individual provider behavior or capacity. The results suggest that workforce structure, including age composition and service infrastructure, may be related to regional disparities in home-visit dental care. Further studies incorporating individual-level and socioeconomic data are needed to clarify underlying mechanisms.

**Supplementary Information:**

The online version contains supplementary material available at https://doi.org/10.1186/s12903-026-08914-2.

## Background

Japan recorded an aging rate of 29.1% as of October 1 st, 2023, one of the highest in the world [1]. Following its peak in 2008, the total population of Japan declined for 11 consecutive years starting in 2011 [1]. Both population aging and population decline in Japan are expected to continue. One of the most notable shifts in medical care resulting from an aging society is the growing demand for home healthcare services. The Ministry of Health, Labour and Welfare (MHLW) estimates that outpatient visits will reach their peak in 2025 and decline thereafter [2].　In contrast, regional demand for home healthcare is projected to rise, leading to a corresponding increase in the need for home-visit dental care. Similar patterns are evident not only in Japan but across multiple countries worldwide [3, 4].

Maintaining optimal oral health is recognized as a critical factor in extending healthy life expectancy, with evidence indicating that oral status has been associated with longevity and risk of requiring long-term care [5, 6]. As a results, improving oral health may contribute to reducing overall healthcare and long-term care expenditures. It is also known that oral health is associated with lifestyle-related diseases and pneumonia [7, 8]. Thus, proper maintenance of oral health is important both socially and personally, and this makes the involvement of dentists indispensable.

Globally, many regions have fewer than five dentists per 10,000 people, and in most countries, the current dental care delivery structures are insufficient to meet the required population needs [9]. In Japan, the number of dentists per 10,000 individuals is 8.4, which is substantial according to global standards [10]. The regional disparities in the provision of home-visit dental care have been observed, influenced by factors like number of dental clinics and dentists, rate of enrollment in college or junior college, and availability of home-visit medical services [11].

International studies have shown that older adults often face barriers to accessing dental care related to accessibility, transportation, workforce availability, and socioeconomic factors [12]. In addition, dentists providing domiciliary dental care may encounter challenges related to time constraints, insufficient training, and limited resources. Home-visit dental care often requires transportation, coordination with community-based care systems, and flexible staffing arrangements, suggesting that regional workforce structure may influence service utilization. In Japan, where population ageing is progressing rapidly, the demand for home-visit dental care is expected to increase. Previous studies have reported regional inequalities in dental care utilization; however, few studies have specifically examined factors associated with regional variation in home-visit dental care utilization, particularly workforce characteristics such as dentist age and clinic availability. The age composition of the dental workforce may reflect broader structural and organizational characteristics of regional dental care systems. We hypothesized that prefectural characteristics related to dental workforce availability and ageing may be associated with regional variation in home-visit dental care utilization. Therefore, this study aimed to examine prefecture-level variation in home-visit dental care SCRs and their associations with dentist workforce characteristics. Because this study was based on prefecture-level aggregated data, the findings should be interpreted as ecological associations rather than individual-level causal relationships.

## Materials and methods

### Data acquisition

To obtain regional dental patient data, we used the 9th edition of the National Database of Health Insurance Claims and Specific Health Checkups of Japan (NDB Open Data), published by the MHLW. The 9th NDB Open Data includes statistics on the number of procedures and patients by disease and care type, based on healthcare insurance claims in Japan from April 2022 to March 2023, and are publicly available [13]. Approximately 236.1 million claims (aggregated records) were included in this study.

Population data for each region were obtained from the publicly available database of “Survey of Population, Population Change, and Number of Households Based on the Basic Resident Registration (as of January 1 st, 2023)” published by the Ministry of Internal Affairs and Communications [14]. Data on per capita prefectural income were obtained from the Prefectural Economic Accounts (FY 2022) published by the Cabinet Office, Government of Japan [15]. Data pertaining to the numbers and average age of dentists and doctors working in medical facilities were obtained from the publicly available database of “Statistics of Physicians, Dentists and Pharmacists, 2022” published by the MHLW [10]. Data regarding the number of dental clinics, medical clinics and hospitals were obtained from the publicly available database “Survey of Medical Institutions, 2022” published by the MHLW [16].

This study used publicly available aggregated data and did not involve individual-level human subjects. Therefore, ethical approval was not required in accordance with national ethical guidelines for studies using publicly available anonymized data.

### Calculation of the standardized claims data ratio

Standardized Claims Data Ratio (SCR), adjusted for sex and five-year age groups, was used to assess the utilization of home-visit dental care and outpatient services (initial and follow-up dental outpatient visit). We used SCR specifically to control for prefectural differences in population composition, thereby reducing confounding by age and sex when comparing regional service utilization. The SCR was calculated using the following formula: [11].

1 $$\begin{aligned} \mathrm{SCR}\:=&\:\{\mathrm\Sigma(\mathrm{number}\;\mathrm{of}\;\mathrm{receipts}\;\mathrm{by}\;\mathrm{age}\;\mathrm{group}) \\ & /\mathrm\Sigma(\mathrm{expected}\;\mathrm{number}\;\mathrm{of}\;\mathrm{receipts}\;\mathrm{by}\;\mathrm{age}\;\mathrm{group})\}\:\times\:100\:\\=&\:\mathrm\Sigma(\mathrm{number}\;\mathrm{of}\;\mathrm{receipts}\;\mathrm{by}\;\mathrm{age}\;\mathrm{group})\\&/\ \mathrm\Sigma(\mathrm{population}\;\mathrm{by}\;\mathrm{age}\;\mathrm{group}\: \times\:\mathrm{national}\;\mathrm{incidence}\\& \mathrm{of}\;\mathrm{receipts}\;\mathrm{by}\;\mathrm{age}\;\mathrm{group})\:\times\:100 \end{aligned}$$

These calculations were performed separately for each prefecture. Based on the SCR values of home-visit dental care, prefectures were classified into four categories (SCR ≥ 150, < 150 and ≥ 100, < 100 and ≥ 50, or < 50) and visually mapped. SCRs for home-visit medical care use publicly available resources from the Cabinet Office [17].　SCR has been used in previous studies to evaluate regional differences in healthcare utilization while adjusting for population age and sex [11]. In this study, SCR was applied to examine regional differences in home-visit dental care, allowing assessment of the relationship between supply-side factors, such as the average age of dentists, and service provision.

### Statistical analyses

This report follows the STROBE guidelines for observational studies. This ecological cross-sectional study was reported in accordance with the STROBE statement. Because this study used aggregated prefecture-level data, only the items relevant to cross-sectional ecological analyses were applied.

**Fig. 1:**
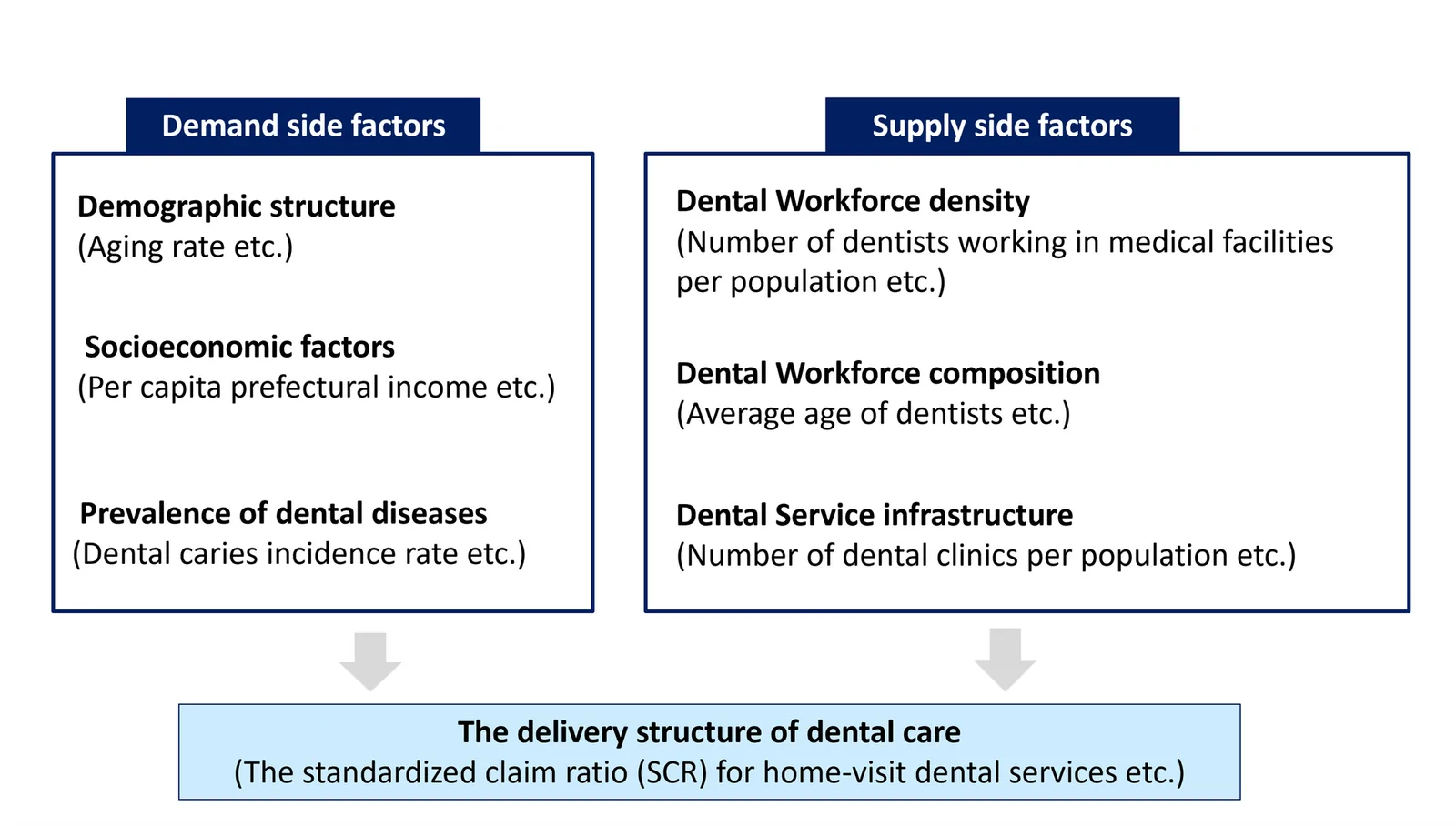
Conceptual framework of factors potentially associated with regional variation in home-visit dental care utilization. The framework distinguishes demand-side factors, including demographic structure (e.g., aging rate) and socioeconomic factors (e.g., income and medical expenditure), from supply-side factors, including dental workforce density (e.g., number of dentists working in medical facilities per population), dental workforce composition (e.g., average age of dentists), and dental service infrastructure (e.g., number of dental clinics per population). These factors were conceptualized as potentially influencing the regional delivery structure of dental care, represented in this study by the standardized claim ratio (SCR) for home-visit dental services. The SCR is interpreted as a composite ecological indicator influenced by both supply- and demand-side factors, rather than a direct measure of provider capacity

This ecological design does not permit inference regarding the clinical behavior or capacity of individual dentists. Based on prior literature and theoretical considerations, we developed a conceptual framework to guide variable selection and model specification (Fig. 1). Indicators of the prevalence of dental diseases were included in the conceptual framework; however, appropriate prefecture-level indicators directly related to the utilization of home-visit dental care among eligible populations were not publicly available and therefore could not be operationalized in the present study. As illustrated in the conceptual framework (Fig. 1), the SCR reflects a composite measure influenced by both supply- and demand-side factors and should not be interpreted as a direct measure of provider capacity. A two-sided *p*-value of < 0.05 was considered statistically significant. All statistical analyses were performed using the IBM SPSS Statistics version 30 (IBM Corp., Armonk, NY, USA).

### Descriptive analyses

Descriptive analyses were conducted for the following prefecture-level indicators: SCR for home-visit dental care, SCR for initial dental outpatient visits, SCR for follow-up dental outpatient visits, aging rate, per capita prefectural income (thousand yen), number of dentists working in medical facilities, number of dental clinics, and average age of dentists. For each variable, the mean, median, standard deviation, minimum, maximum, and variance were calculated. The numbers of dentists working in medical facilities and dental clinics were standardized by prefectural population and expressed per 100,000 population.

### Correlation analyses

Because the prefecture-level variables were not assumed to follow a normal distribution and the sample size was relatively small (47 prefectures), non-parametric methods were used.　Correlations were assessed between the SCR for home-visit dental care and the following prefecture-level indicators: SCR for initial dental outpatient visits, SCR for follow-up dental outpatient visits, aging rate (percentage of the population aged ≥ 65 years), per capita prefectural income (thousand yen), number of dentists working in medical facilities (including privately owned dental clinics), number of dental clinics, and average age of dentists. The SCRs for initial and follow-up dental outpatient visits were included as contextual indicators reflecting general dental service utilization. The aging rate and was analyzed as a proxy indicator of population aging–related demand.

### Regression analyses

Based on prior literature and theoretical considerations, prefecture-level variables reflecting demographic structure and dental workforce characteristics were selected for analysis. In the primary multivariable regression model, the SCR for home-visit dental care was specified as the dependent variable. Independent variables included average dentist age, number of dental clinics per 100,000 population, and regional aging rate. The regional aging rate was included as a proxy indicator of demographic demand, whereas the number of dental clinics per 100,000 population was included as an indicator of dental service availability. Average dentist age was included as a workforce composition indicator. The numbers of dentists and dental clinics were standardized by prefectural population and expressed per 100,000 population. Because the number of dentists and the number of dental clinics were conceptually and statistically highly correlated, only the number of dental clinics was retained in the final multivariable model to reduce multicollinearity.

### Exploratory medical-care analyses

First, descriptive analyses were conducted to examine prefecture-level variation in SCRs for home-visit dental and medical care. Prefecture-level indicators analyzed in both dental and medical care settings included SCRs for home-visit dental and medical care, provider numbers, clinic numbers, and average provider age. Second, Wilcoxon rank sum test were used to compare paired distributions of these indicators between dental and medical care as contextual reference analyses. Third, Spearman’s rank correlation coefficient analyses were conducted to assess associations among these indicators and home-visit care SCRs. Analyses involving home-visit medical care variables were conducted separately as exploratory contextual comparisons to evaluate whether broader regional patterns observed in home-visit dental care were also seen in medical care; these analyses were not intended as primary study outcomes or adjustment variables in the dental care regression models.

## Results

**Fig. 2:**
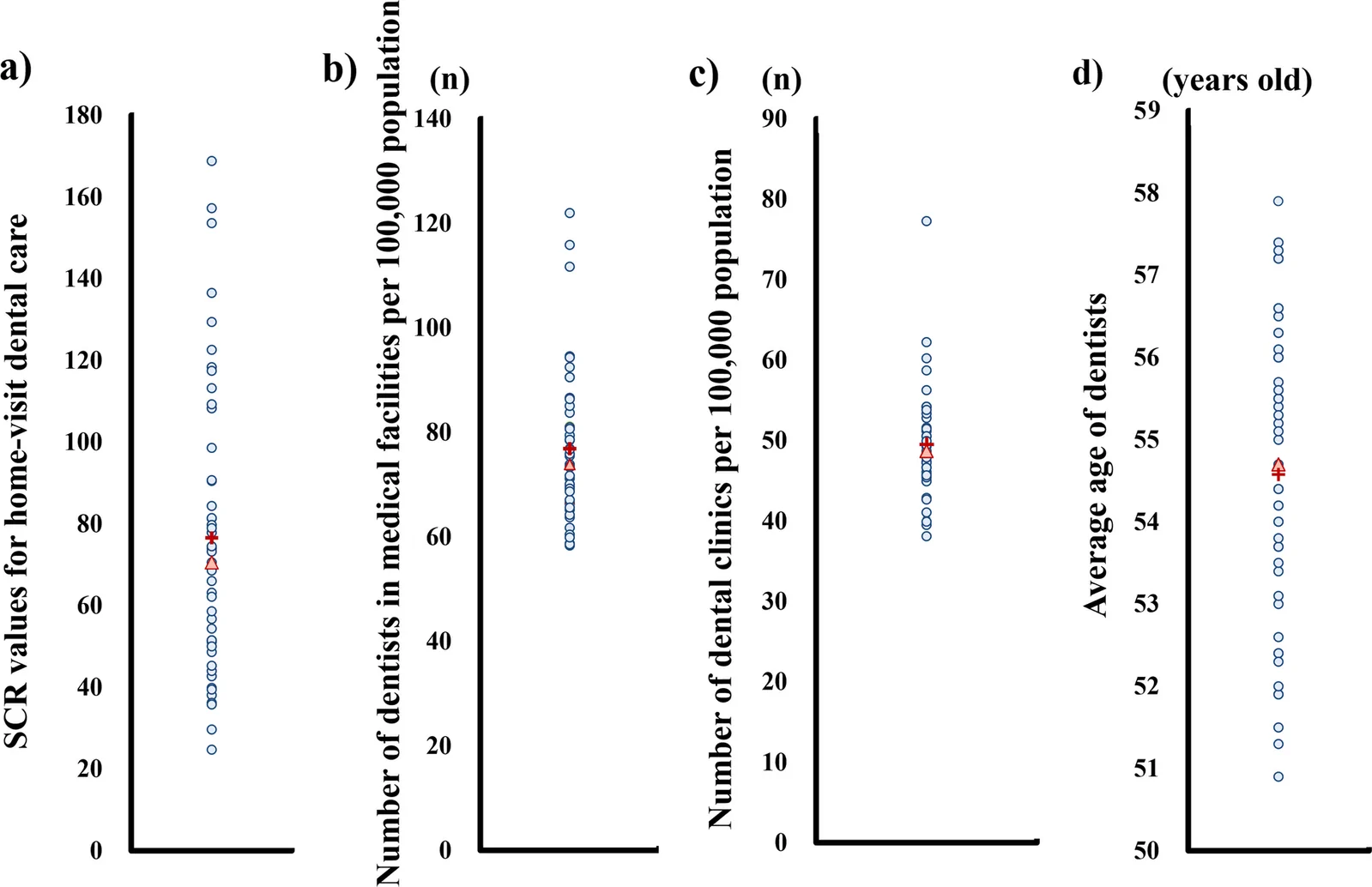
Scatter Plots of Each Indicator by Prefecture. Each dots indicate an individual prefecture. The red triangles indicate the median values, while the red crosses represent the mean values. **a** Standardized Claims Data Ratio of home-visit dental care. **b** Number of dentists working in medical facilities per 100,000 population. **c** Number of dental clinics per 100,000 population. **d** Average age of dentists. The numbers of dentists working in medical facilities and dental clinics were standardized by prefectural population and expressed per 100,000 population. Sample size: *N* = 47 prefectures

Basic statistical data extracted from each database, along with SCR of home-visit dental care and medical care, are presented in Table 1 and Supplementary Table 1, and their distribution is shown in Fig. 2 and supplementary Fig. 2, respectively. Across 47 prefectures, the SCR for home‑visit dental care ranged from 24.68 to 168.79 (median 70.52; mean 76.60 ± 35.74). Other variables were summarized as follows (mean ± standard deviation): the SCR value of the initial dental outpatient visits, 96.04 ± 10.01; the SCR value of the follow-up dental outpatient visits, 97.62 ± 6.76; aging rate, 30.76 ± 3.16; per capita prefectural income (thousand yen), 3079.23 ± 541.46; number of dentists working in medical facilities per 100,000 population, 76.85 ± 14.41; number of dental clinics per 100,000 population, 49.54 ± 6.65; and average age of dentists, 54.57 ± 1.84 (Table 1). For the home-visit medical care, the SCR value of the home-visit medical care, the SCR value of the initial medical outpatient visits, the SCR value of the follow-up medical outpatient visits, aging rate, per capita prefectural income (thousand yen), number of doctors working in medical facilities per 100,000 population, number of medical clinics per 100,000 population, number of hospitals per 100,000 population and average age of doctors were 82.89 ± 28.18, 98.35 ± 7.94, 100.21 ± 7.94, 30.76 ± 3.16, 3079.23 ± 541.46, 265.90 ± 40.19, 84.11 ± 12.06, 7.95 ± 3.14, and 51.13 ± 1.37, respectively (Supplementary Table 1). Number of dentists or doctors working in medical facilities, number of clinics and average age were significantly different between the two groups (*p* < 0.01). However, the SCR values between two groups were not significantly different.

**Table 1:** Summary statistics of the standardized claims data ratio for home-visit dental care and associated indicators

	SCR^a^ of home-visit dental care	SCR^a^ of initial dental outpatient visits	SCR^a^ of follow-up dental outpatient visits	Aging rate	Per capita prefectural income (thousand yen)	Number of dentists working in medical facilities	Number of dental clinics	Average age of dentists
Mean	76.60	96.04	97.62	30.76	3079.23	76.85	49.54	54.57
Median	70.52	96.74	97.98	31.03	3031.00	73.85	48.68	54.70
Standard deviation	35.74	10.01	6.76	3.16	541.46	14.41	6.65	1.84
Minimum	24.68	75.31	79.29	22.79	2,249	58.36	38.11	50.9
Maximaum	168.79	112.80	113.72	37.78	6,037	122.02	77.27	57.9
Variance	1277.48	100.23	45.64	10.02	293,179.27	207.67	44.21	3.39

The numbers of dentists working in medical facilities and dental clinics were standardized by prefectural population and expressed per 100,000 population

^a^Standardized Claims Data Ratio

Sample size: *N* = 47 prefectures

**Fig. 3:**
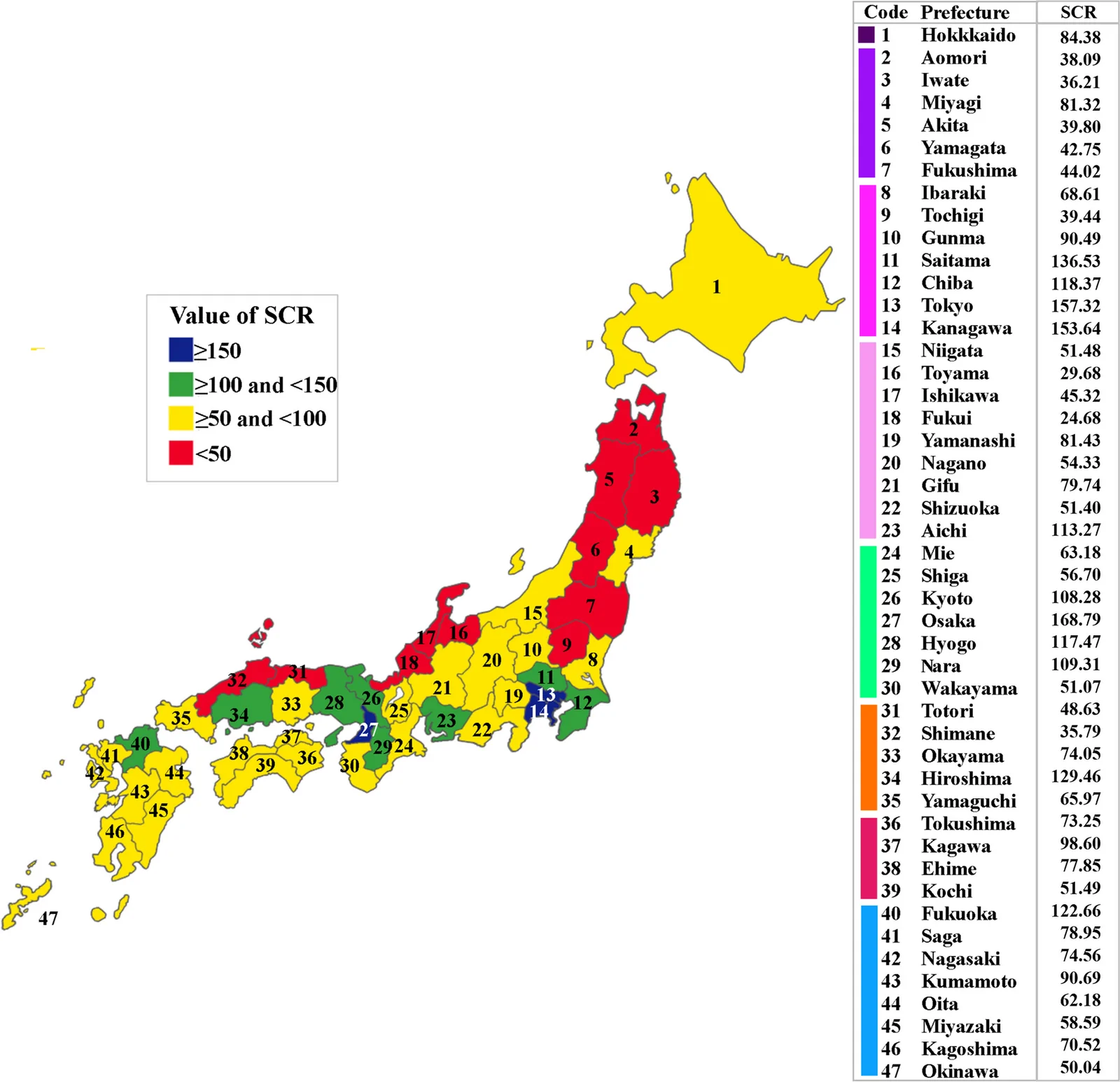
Map of 47 prefectures categorized on the standardized claims data ratio of home-visit dental care. Each prefecture on the map is categorized into four groups based on its value of the standardized claims data ratio of home-visit dental care, represented by color: blue indicates a value of 150 or higher, green indicates a value of 100 to less than 150, yellow indicates a value of 50 to less than 100, and red indicates a value of less than 50. The numbers assigned to each prefecture correspond to the prefecture codes listed in the table on the right side of the figure. The colors adjacent to each prefecture name in the code table represent the geographic regions of Japan.: dark purple represent the Hokkaido region; purple represent the Tohoku region, dark pink represent the Kanto region, light pink represent the Chubu region, Light green represent the Kansai region, orange represent the Chugoku region, Magenta represent the Shikoku region, and light blue represent the Kyushu and Okinawa region. SCR; standardized claims data ratio

SCR values of home-visit dental care for each prefecture were classified into four SCR categories (SCR ≥ 150, < 150 and ≥ 100, < 100 and ≥ 50, or < 50) and visualized using color- coded map (Fig. 3). Notably, prefectures with major metropolitan areas, such as Tokyo, Kanagawa, and Osaka, showed SCR value exceeding 150. In contrast, several prefectures recorded SCR values below 50, including those in the Tohoku region (Aomori, Iwate, Akita, Yamagata, Fukushima), the Hokuriku region (Toyama, Ishikawa, Fukui), Tochigi in the Kanto region, and Tottori and Shimane in the Chugoku region.

Table 2 and Supplementary Table 2 summarize the results of the correlation analysis. For home-visit dental care, the SCR of dental home-visit care showed a significant positive correlation with number of dentists working in medical facilities per 100,000 population (R = 0.60, *p* < 0.001) and the number of dental clinics per 100,000 population (R = 0.71, *p* < 0.001). In contrast, a negative correlation was found between the SCR for home-visit dental care and the average age of dentists (R = − 0.65, *p* < 0.001). Additionally, the average age of dentists was negatively correlated with both the number of dentists working in medical facilities per 100,000 population (R = − 0.72, *p* < 0.001) and the number of dental clinics per 100,000 population (R = − 0.47, *p* < 0.001). The average age of dentists was also negatively correlated with the SCR of initial dental outpatient visits (R = − 0.31, *p* < 0.05) and the SCR of follow-up dental outpatient visits (R = − 0.34, *p* < 0.05); however, these correlations were weaker than that observed for the SCR of home-visit dental care. Per capita prefectural income (thousand yen), which was considered a representative demand-side factor, was not significantly correlated with the SCR of home-visit dental care, whereas the aging rate, also considered a representative demand-side factor, showed a moderate negative correlation (R = − 0.57, *p* < 0.001). For the home-visit medical care, the SCR of home-visit medical care showed a positive correlation with the number of medical clinics per 100,000 population (R = 0.32, *p* < 0.05). Similar to the dental care findings, the average age of doctors showed a negative correlation with the SCR for home-visit dental care (R = − 0.33, *p* < 0.05). There was a positive correlation between the average age of doctors and the SCR of follow-up medical outpatient visits (R = 0.31, *p* < 0.05). Furthermore, similar to the findings for dental care, the aging rate showed a moderate negative correlation with the SCR of home-visit medical care (R = − 0.53, *p* < 0.001). In contrast, per capita prefectural income (thousand yen) showed a weak positive correlation (R = 0.37, *p* < 0.05), which differed from the findings for dental care.

**Table 2:** Spearmanʼs correlation coefficient analysis among associated indicators

	SCR^a^ of home-visit dental care	SCR^a^ of initial dental outpatient visits	SCR^a^ of follow-up dental outpatient visits	Aging rate	Per capita prefectural income (thousand yen)	Number of dentists working in medical facilities	Number of dental clinics	Average age of dentists
SCR^a^ of dental home-visit care	-							
SCR^a^ of initial dental outpatient visits	0.41^c^	-						
SCR^a^ of follow-up dental outpatient visits	0.47^b^	0.17^e^	-					
Aging rate	− 0.57^b^	− 0.53^b^	− 0.38^c^	-				
Per capita prefectural income (thousand yen)	0.24^e^	0.43^c^	0.44^c^	− 0.46 ^c^	-			
Number of dentists working in medical facilities	0.60^b^	0.16^e^	0.37^d^	− 0.19^e^	0.03^e^	-		
Number of dental clinics	0.71^b^	0.39^c^	0.33^d^	− 0.23^e^	0.22^e^	0.76^b^	-	
Average age of dentists	− 0.65^b^	− 0.31^d^	− 0.34^d^	0.61^b^	− 0.10^e^	− 0.72^b^	− 0.47^b^	-

The numbers of dentists working in medical facilities and dental clinics were standardized by prefectural population and expressed per 100,000 population

Sample size: *N* = 47 prefectures

^a^Standardized Claims Data Ratio

^b^*p* < 0.001; Spearmanʼs correlation coefficient

^c^*p* < 0.01; Spearmanʼs correlation coefficient

^d^*p* < 0.05; Spearmanʼs correlation coefficient

^e^*p* > 0.05; Spearmanʼs correlation coefficient

Table 3 present the results of the multiple regression analysis examining the relationship of each factor on SCR of dental home-visit care, adjusted R^2^ = 0.68). The average age of dentists showed a negative association with the SCR for dental home-visit care (the standardized regression coefficient (β) = − 0.30, *p* = 0.017) and the aging rate (the standardized regression coefficient (β) = − 0.27, *p* = 0.018), while the number of dental clinics per 100,000 population showed a positive effect (β = 0.47, *p* < 0.001). For SCR for medical home visit care, adjusted R^2^ value was 0.52. The number of medical clinics per 100,000 population showed a positive association (β = 0.45, *p* < 0.001). The aging rate showed a negative association with the SCR for medical home visit care (the standardized regression coefficient (β) = − 0.65, *p* < 0.001) The average age of doctors did not show a statistically significant association with the SCR for medical home visit care (β = − 0.05, *p* = 0.730).

**Table 3:** Multiple regression analysis of the standardized claims data ratio for home-visit dental care

	B ^a^	Standard error	β^b^	*t*	*p*	95% CI ^c^ for B^a^	VIF ^d^
(Constant)	357.55	123.23	-	2.90	0.006	109.03 −606.07	
Number of dental clinics	2.52	0.52	0.47	4.84	< 0.001	1.47—3.57	1.34
Average age of dentists	− 5.74	2.32	− 0.30	− 2.48	0.017	− 10.41—− 1.07	2.04
Aging rate	− 3.01	1.23	− 0.27	− 2.45	0.018	− 5.49—− 0.54	1.69

The numbers of dental clinics were standardized by prefectural population and expressed per 100,000 population

Sample size: *N* = 47 prefectures; R^2^ = 0.70; Adjusted R^2^ = 0.68

^a^Unstandardized Coefficients

^b^Standardized Coefficients

^c^Confidence Interval

^d^Variance Inflation Factor

## Discussion

In the present study, we identified regional variation in home-visit dental care utilization across Japan and observed associations with dental workforce characteristics, including average dentist age and the number of dental clinics. However, given the ecological study design using prefecture-level data, these findings should be interpreted as area-level associations rather than individual-level effects.

Home-visit dental care plays a critical role in supporting public health. A substantial number of older individuals requiring long-term medical care need dental care, and in many cases, medical conditions necessitate the use of home-visit dental services [18]. Furthermore, the global emphasis on strengthening home healthcare systems highlights the need for the expansion of such service [3, 4]. In addition, oral health has been implicated in systemic diseases, including diabetes, cardiovascular disease, dementia, respiratory disease, and cancer [7, 19–21]. Accordingly, sustaining the availability of dental care at the community level is crucial to promoting and maintaining the public health.

There was considerable variation in the basic statistical data among the prefectures (Table 1 and Fig. 2). Interestingly, the SCR for home-visit dental care, the number of dentists working in medical facilities, and the number of dental clinics exhibited similar distribution patterns. The average age of dentists was approximately evenly distributed between 50.80 and 57.90 years. Although these prefectural-level statistics suggest potential regional disparities, firm conclusions cannot be drawn solely from this data. Further investigation using geospatial or more granular analyses is warranted. In addition, the analysis in this study was limited to the year for which synchronized data on patient numbers, number of dentists, and average age of dentists were available, and future studies using synchronized datasets across multiple years would allow for more robust temporal analyses.

This study found that the number of dental clinics and dentists was significantly associated with the SCR of dental home-visit care (Table 2). It was also suggested that the provision of dental home-visit services tended to be relatively limited in regions such as Tohoku and Hokuriku region. A previous study reported that significant regional disparities in periodontal care and home-visit dental care, influenced by factors such as the number of dental clinics, income levels, and educational attainment [11]. These findings indicate that dental home-visit services are more developed in urban regions, whereas rural areas may face disparities in access to these services. In addition to the limited number of dentists, factors such as transportation accessibility and climate may also contribute to the low provision of dental home-visit care in the Tohoku region [16, 22]. Furthermore, disparities were also observed within individual prefectures, suggesting that the actual situation may vary even within the same prefecture. Therefore, future research should incorporate more granular and multifaceted analyses at smaller administrative levels, such as municipalities or secondary medical areas.

In this study, we found a correlation between the numbers of dental clinics and dentists working in medical facilities and the SCRs for home-visit dental care (Table 2) and the results of multiple regression analysis suggested the number of dental clinics had a positive association with the provision system of home-visit dental care (Table 3). These interpretations are consistent with the visually assessed linearity shown in Supplementary Fig. 1. The adequacy of the linear model was supported by the visual inspection of scatter plots with fitted regression lines (Supplementary Fig. 1). Increasing the number of dentists and dental clinics may represent one potential consideration when discussing how to strengthen the provision system for home-visit dental services. However, in recent years, the numbers of dentists and dental clinics in Japan have shown a declining trend [10, 16]. Given their uneven distribution, particularly the concentration of dentists in urban areas, it is imperative to evaluate contributing factors and consider potential solutions. It should be noted that SCR may not solely reflect the availability of home-visit dental care, but also regional demand factors, such as population age structure, prevalence of disability, long-term care insurance certification rates, and nursing home density. Socioeconomic factors are considered to influence the utilization of home-visit dental care. Per capita prefectural income (thousand yen) was not significantly correlated with the SCR of home-visit dental care. In Japan, financial barriers to access may be partially mitigated by the universal health insurance system and social security programs, including public pension schemes, public assistance (welfare), and disability support. However, these mechanisms do not eliminate regional or individual-level socioeconomic differences. Therefore, residual confounding by socioeconomic factors cannot be ruled out. Therefore, interpretation of SCR should consider potential confounding from these demand-side factors. However, given the observed negative correlation between the aging rate and the SCRs for home-visit dental care, it is possible that, in some regions, the supply of home-visit dental services does not adequately meet the growing demand associated with population aging.

Furthermore, efforts should be made to increase the number of dental institutions offering home-visit dental care. According to a survey conducted by the MHLW, only 33.9% of dental clinics provided dental home-visit care in 2023 [23], meaning that over 60% did not. In addition, the average number of dentists per dental clinic in Japan was 1.5 in the fiscal year 2023 [24]. Since providing both outpatient and home-visit dental care requires multiple dentists, clinics with a limited staff face challenges in providing home-visit dental care. Dental clinics that meet certain performance criteria providing home-visit dental care are officially designated as “Home Care Supporting Dental Clinics” in Japan. However, the average number of dentists in these designated dental clinics is only 1.8, which further suggests the inherent difficulty of sustaining and providing home-visit dental care in Japan [25]. These findings suggest that the number of dentists and dental clinics is a critical factor in expanding the scope of home-visit dental care.

More importantly, the average age of dentists in a given region was negatively associated with both the SCR for home-visit dental care and the number of dentists and dental clinics (Table 2). Notably, the average age of dentists showed a stronger negative correlation with the SCR for home-visit dental care than with the SCRs for outpatient dental care, suggesting a potentially stronger association with home-visit services than with outpatient services (Table 2). Although this interpretation remains speculative and cannot be verified within the scope of this ecological analysis, several plausible mechanisms may explain this association. One possible explanation is that regions with an older dental workforce may be characterized by structural or organizational constraints that limit the provision of home-visit dental care. These potential mechanisms are presented as ecological interpretations and do not imply individual-level provider behavior. Since the provision of home-visit dental care involves traveling to patients’ homes and working in less well-equipped environments, regions with an older dental workforce may face structural or logistical challenges in sustaining such services. Moreover, these services often require coordination, mobility, and resource allocation at the system level, which may be influenced by regional workforce composition. The scarcity of facilities in dentistry capable of attracting young dentists, such as hospitals, is considered one factor contributing to the influence of dentist age on the regional dental care delivery system. If the current workforce trends continue, they may be associated with a reduction in regional service capacity. In addition to dentist age and clinic availability, other workforce characteristics may also influence the provision of home-visit dental care. For example, gender distribution, practice type (e.g., clinic-based or hospital-affiliated practice), and overall workforce composition may affect the capacity and willingness to provide home-based services. However, these variables were not available in the present ecological dataset and should be considered in future research.

The analysis of home-visit medical care revealed similar structural challenges (supplementary Fig. 2 and supplementary Table 1). Although the SCR values for home-visit care between dental and medical services were not significantly different (Wilcoxon signed-rank test, *p* = 0.077), the number of doctors, clinics, and hospitals varied widely across regions. The SCR for medical care was positively associated with the number of medical clinics, while no significant association was observed with the number of doctors or hospitals. In contrast, the SCR was negatively associated with the average age of doctors (supplementary Table 2). These findings suggest that, at the unadjusted level, both workforce age composition and clinic distribution may be related to regional variation in home-visit medical service utilization. However, in the multivariable regression analysis, the positive association between medical SCR and the number of medical clinics remained statistically significant, whereas the association with the average age of doctors was attenuated and no longer statistically significant after adjustment (Supplementary Table 3). This attenuation may reflect the influence of collinearity among workforce-related variables or shared underlying regional structural factors. Interestingly, the number of medical clinics showed a robust positive association with medical SCR in the multivariable model. This finding suggests that the density of medical clinics may play an important role in supporting the provision of home-visit medical care, highlighting the relevance of clinic availability in regional healthcare delivery capacity. Overall, these findings suggest that the provision of home-visit medical services may be more closely linked to the distribution of clinic-based care infrastructure than to overall physician supply or age composition. However, these differences should be interpreted cautiously, given the ecological nature of the data and potential residual confounding. In contrast to dental care, per capita prefectural income showed a weak positive association with home-visit medical care, which may indicate differences in how socioeconomic conditions influence access to medical versus dental home-visit services. This discrepancy may be attributable to differences in service delivery models, provider availability, or patient characteristics between medical and dental care systems.

This study suggests that regional differences in home-visit dental care utilization are associated with structural characteristics of the dental workforce, including the distribution of dental clinics and the average age of dentists. Notably, regions characterized by an older dental workforce tended to show lower levels of home-visit dental care utilization. However, these findings should be interpreted at the ecological level and do not imply causal relationships at the individual dentist level. The parallel analyses of medical care provide contextual information on workforce structure and facility distribution across healthcare domains, rather than indicating direct equivalence between dental and medical home-visit care systems. These findings may offer descriptive information that could inform discussions on strategies to address regional disparities and improve access to home-visit healthcare, including dental and medical services, within community-based care systems. However, further studies using individual-level data are required to clarify causal mechanisms and underlying determinants.

### Limitations

This study has some limitations that should be considered.

1. Ecological study design: This study used prefecture-level aggregated data, and individual-level causal inference is not possible. Regional heterogeneity within prefectures, including local service availability and patient characteristics, could not be captured.
2. Incomplete adjustment for demand and supply factors: Although the aging rate was included as a proxy for demographic demand, other demand-related factors (e.g., disability prevalence, long-term care certification rates, infrastructure, and nursing home density) were not fully adjusted for. Moreover, the Standardized Claims Data Ratio (SCR) reflects realized utilization and is influenced by both supply and demand conditions; thus, it does not directly measure service capacity or supply–demand balance. Importantly, direct equilibrium metrics (e.g., unmet need, waiting times, or capacity/utilization measures) were unavailable in the publicly released aggregated datasets and therefore could not be incorporated, and SCR should not be interpreted as a direct measure of provider capacity. In addition, while SCR facilitates demographic standardization, it is not intended to analyze age‑ or sex‑specific patterns themselves; such analyses would require non‑standardized strata.
3. Workforce structure and cohort effects: Although a negative association was observed between the average age of dentists and home-visit dental care utilization, we could not evaluate clinic establishment year or cohort‑specific practice patterns; residual confounding by generational or organizational factors cannot be excluded. Accordingly, the observation relationship between dentist age and service provision should be interpreted with caution as an ecological association rather than an individual-level effect.
4. Region‑specific clinical and service characteristics: This ecological analysis could not capture intra‑prefectural heterogeneity in clinical needs or service organization. Potentially relevant factors include the prevalence and severity of oral diseases, multimorbidity patterns, the density and distribution of long‑term care facilities, municipality‑level outreach programs, availability of home‑visit equipment and mobile dental units, and local care pathways (e.g., hospital‑based vs. clinic‑based coordination). Because the publicly released aggregated datasets provide prefecture‑level summaries only, these micro‑level service characteristics were not available. Consequently, the observed associations at the prefecture level may partly reflect unmeasured variation in local clinical profiles and service delivery models, and should be interpreted with appropriate caution.
5. Possible cross-prefecture service flows: Service provision across prefectural borders may have influenced SCR values.

## Supplementary Information


Additional file 1.
Additional file 2.
Additional file 3.


## Data Availability

The datasets using this study are available from the Ministry of Health, Labour and Welfare, the Cabinet Office and the Ministry of internal affairs and communications of the Japanese government website.
