# Supplementary material for: Prefecture-level factors associated with home-visit dental care utilization in Japan: focus on dentist workforce characteristics
Source: BMC Oral Health. 2026 Jun 20;26:1635. doi: 10.1186/s12903-026-08914-2 (PMC13528153; doi:10.1186/s12903-026-08914-2)
Supplement: Supplementary file 1 [file 12903_2026_8914_MOESM3_ESM.docx]

**Supplement Figure Legends**

**Supplement Figure 1**

**Scatter plots showing the relationship between predictor variables and the standardized claims ratio (SCR) for home-visit dental care.**

Scatter plots illustrating the associations between the SCR for home-visit dental care and a) average age of dentists, b) number of dental clinics per 100,000 population, and c) regional aging rate (percentage of population aged ≥65 years) across prefectures. A linear regression line is included in each plot to visually assess the linearity of the relationships.

The numbers of dental clinics were standardized by prefectural population and expressed per 100,000 population.

**Supplement Figure 2**

**Scatter Plots of Standardized Claims Data Ratio of home-visit medical care and Each Indicator by Prefecture.**

Each dot indicates an individual prefecture. The red triangles indicate the median values, while the red crosses represent the mean values. **a)** Standardized Claims Data Ratio of home-visit medical care. **b)** Number of doctors working in medical facilities per 100,000 population. **c)** Number of medical clinics per 100,000 population. **d)** Number of hospitals per 100,000 population **e)** Average age of doctors.

The numbers of doctors working in medical facilities, medical clinics and hospitals were standardized by prefectural population and expressed per 100,000 population.

Sample size: N = 47 prefectures.

**Supplement table**

**Table 1. Summary statistics of the standardized claims data ratio for home-visit medical** care **and associated indicators.**

|  | SCR^a^ of home-visit medical care | SCR^a^ of initial medical outpatient visits | SCR^a^ of follow-up medical outpatient visits | Aging rate | Per capita prefectural income (thousand yen) | Number of doctors working in medical facilities | Number of medical clinics | Number of hospitals | Average age of doctors |
| --- | --- | --- | --- | --- | --- | --- | --- | --- | --- |
| Mean | 82.89 | 98.35 | 100.21 | 30.76 | 3079.23 | 265.90 | 84.11 | 7.95 | 51.13 |
| Median | 79.20 | 98.40 | 102.20 | 31.03 | 3031.00 | 264.52 | 85.63 | 6.98 | 51.10 |
| Standard deviation | 28.18 | 7.94 | 7.94 | 3.16 | 541.46 | 40.19 | 12.06 | 3.14 | 1.37 |
| Minimum | 37.1 | 83.4 | 78.3 | 22.79 | 2,249 | 179.16 | 60.90 | 3.65 | 48.2 |
| Maximaum | 158.3 | 114.5 | 116.6 | 37.78 | 6,037 | 340.84 | 111.44 | 17.53 | 54.2 |
| Variance | 793.97 | 63.01 | 63.05 | 10.02 | 293,179.27 | 1615.10 | 145.37 | 9.89 | 1.89 |

^a^Standardized Claims Data Ratio

Sample size: N = 47 prefectures.

The numbers of doctors working in medical facilities, medical clinics and hospitals were standardized by prefectural population and expressed per 100,000 population.

**Supplement table 2.**

**Spearmanʼs correlation coefficient analysis among the standardized claims data ratio for home-visit medical care and related indicators.**

|  | SCR^a^ of home-visit medical care | SCR^a^ of initial medical outpatient visits | SCR^a^ of follow-up medical outpatient visits | Aging rate | Per capita prefectural income (thousand yen) | Number of doctors working in medical facilities | Number of medical clinics | Number of hospitals | Average age of doctors |
| --- | --- | --- | --- | --- | --- | --- | --- | --- | --- |
| SCR^a^ of home-visit medical care | - |  |  |  |  |  |  |  |  |
| SCR^a^ of initial medical outpatient visits | 0.49^b^ | - |  |  |  |  |  |  |  |
| SCR^a^ of follow-up medical outpatient visits | 0.14^e^ | 0.49^b^ | - |  |  |  |  |  |  |
| Aging rate | −0.53^b^ | −0.22^e^ | 0.16^e^ | - |  |  |  |  |  |
| Per capita prefectural income (thousand yen) | 0.37^d^ | 0.00^e^ | 0.01^e^ | −0.46^c^ | - |  |  |  |  |
| Number of doctors working in medical facilities | 0.22^e^ | 0.53^b^ | 0.24^e^ | 0.17^e^ | −0.27^e^ | - |  |  |  |
| Number of medical clinics | 0.32^d^ | 0.54^b^ | 0.50^b^ | 0.19^e^ | −0.01^e^ | 0.71^b^ | - |  |  |
| Number of hospitals | −0.26^e^ | 0.27^e^ | 0.21^e^ | 0.65^b^ | −0.50^b^ | 0.56^b^ | 0.28^e^ | - |  |
| Average age of doctors | −0.33 ^d^ | 0.09^e^ | 0.31^d^ | 0.71^b^ | −0.39^c^ | 0.17^e^ | 0.24^e^ | 0.70^b^ | - |

^a^Standardized Claims Data Ratio

^b^*p* <0.001; Spearmanʼs correlation coefficient

^c^*p* <0.01; Spearmanʼs correlation coefficient

^d^*p* <0.05; Spearmanʼs correlation coefficient

^e^*p* >0.05; Spearmanʼs correlation coefficient

Sample size: N = 47 prefectures.

The numbers of doctors working in medical facilities, medical clinics and hospitals were standardized by prefectural population and expressed per 100,000 population.

**Supplement table 3. Multiple regression analysis of the standardized claims data ratio for home-visit medical care.**

|  | B ^a^ | Standard error | β^b^ | *t* | *p* | 95% CI ^c^　for B^a^ | VIF ^d^ |
| --- | --- | --- | --- | --- | --- | --- | --- |
| (Constant) | 225.46 | 126.29 | - | 1.79 | 0.081 | −29.23 -  480.14 |  |
| Number of medical clinics | 1.05 | 0.25 | 0.45 | 4.25 | ＜0.001 | 0.55 - 1.55 | 1.06 |
| Average age of doctors | −1.03 | 2.97 | −0.05 | −0.35 | 0.73 | −7.01 - 4.96 | 1.99 |
| Aging rate | −5.79 | 1.28 | −0.65 | −4.52 | ＜0.001 | −8.38 - −3.21 | 1.97 |

^a^ Unstandardized Coefficients

^b^ Standardized Coefficients

^c^ Confidence Interval

^d^ Variance Inflation Factor

Sample size: N = 47 prefectures; R^2^ = 0.55; Adjusted R^2^ = 0.52

The numbers of medical clinics were standardized by prefectural population and expressed per 100,000 population.
